# Potential Mitochondrial Isocitrate Dehydrogenase R140Q Mutant Inhibitor from Traditional Chinese Medicine against Cancers

**DOI:** 10.1155/2014/364625

**Published:** 2014-06-05

**Authors:** Wen-Yuan Lee, Kuan-Chung Chen, Hsin-Yi Chen, Calvin Yu-Chian Chen

**Affiliations:** ^1^Department of Biomedical Informatics, Asia University, Taichung 41354, Taiwan; ^2^School of Medicine, College of Medicine, China Medical University, Taichung 40402, Taiwan; ^3^Department of Neurosurgery, China Medical University Hospital, Taichung 40447, Taiwan; ^4^School of Pharmacy, China Medical University, Taichung 40402, Taiwan; ^5^Research Center for Chinese Medicine & Acupuncture, China Medical University, Taichung 40402, Taiwan

## Abstract

A recent research of cancer has indicated that the mutant of isocitrate dehydrogenase 1 and 2 (*IDH1* and *2*) genes will induce various cancers, including chondrosarcoma, cholangiocarcinomas, and acute myelogenous leukemia due to the effect of point mutations in the active-site arginine residues of isocitrate dehydrogenase (IDH), such as IDH1/R132, IDH2/R140, and IDH2/R172. As the inhibition for those tumor-associated mutant IDH proteins may induce differentiation of those cancer cells, these tumor-associated mutant IDH proteins can be treated as a drug target proteins for a differentiation therapy against cancers. In this study, we aim to identify the potent TCM compounds from the TCM Database@Taiwan as lead compounds of IDH2 R140Q mutant inhibitor. Comparing to the IDH2 R140Q mutant protein inhibitor, AGI-6780, the top two TCM compounds, precatorine and abrine, have higher binding affinities with target protein in docking simulation. After MD simulation, the top two TCM compounds remain as the same docking poses under dynamic conditions. In addition, precatorine is extracted from *Abrus precatorius* L., which represents the cytotoxic and proapoptotic effects for breast cancer and several tumor lines. Hence, we propose the TCM compounds, precatorine and abrine, as potential candidates as lead compounds for further study in drug development process with the IDH2 R140Q mutant protein against cancer.

## 1. Introduction

Nowadays, in accordance with more and more mechanisms of diseases being identified [1–6], there are increasing numbers of potential target proteins against each disease, which are useful for drug design [7–11]. The recent research of cancer has indicated that the mutant of isocitrate dehydrogenase 1 and 2 (*IDH1* and* 2*) genes will induce various cancers [12, 13]. Somatic mutations in the isocitrate dehydrogenase 1 and 2 genes affecting point mutations in the active-site arginine residues of isocitrate dehydrogenase (IDH), such as IDH1/R132, IDH2/R140, and IDH2/R172, occur frequently in many cancers, including chondrosarcoma, cholangiocarcinomas, and acute myelogenous leukemia [14–22]. The inhibition for those tumor-associated mutant IDH proteins may induce differentiation of those cancer cells. The tumor-associated mutant IDH proteins can be treated as a drug target proteins for a differentiation therapy against cancers [23].

Nowadays, the computer-aided drug design has been widely used in drug designing [24, 25]. Increasing numbers of compounds extracted from traditional Chinese medicine (TCM) have been indicated as potential lead compounds against cancers [26–28], inflammation [29], influenza [30], viral infection [31], metabolic syndrome [32], diabetes [33], stroke [34–36], and many other diseases [37–41]. A recent research of mutant IDH2 protein shows a compound, AGI-6780, which can inhibit the tumor-associated mutant IDH2/R140Q [42]. For drug development of TCM compounds, we aim to identify the potent TCM compounds from the TCM Database@Taiwan [43] as lead compounds of IDH2 R140Q mutant inhibitor. As structural disordered disposition in the protein may induce the side effect and reduce the occupancy for ligand to bind with target protein [44, 45], PONDR-Fit protocol was performed to predict the disordered disposition in IDH2 protein before virtual screening. After virtual screening, the MD simulation was performed to validate the stability of interactions between IDH2 R140Q mutant proteins and each ligand.

## 2. Materials and Methods

### 2.1. Data Collection

The X-ray crystallography structure of the human mitochondrial isocitrate dehydrogenase (IDH2) R140Q mutant was downloaded from RCSB Protein Data Bank with PDB ID: 4JA8 [42]. To predict the disordered amino acids, PONDR-Fit [46] protocol was employed with the sequence of IDH2 protein from Swiss-Prot (UniProtKB: P48735). In preparation section, X-ray crystallography structure of IDH2 R140Q mutant protein was protonated with Chemistry at HARvard Macromolecular Mechanics (CHARMM) force field [47] and removed crystal water by Prepare Protein module in Discovery Studio 2.5 (DS2.5). The final structure of TCM compounds from TCM Database@Taiwan [43] was protonated and filtered by Lipinski's Rule of Five [48] using Prepare Ligand module in DS2.5. The binding site for virtual screening was defined by the volume of the cocrystallized IDH2 R140Q mutant inhibitor, AGI-6780.

### 2.2. Docking Simulation

The TCM compounds were docking into the binding site using a shape filter and Monte-Carlo ligand conformation generation by LigandFit protocol [49] in DS 2.5. The docking poses were optionally minimized with CHARMM force field [47] and filtered the similar poses by the clustering algorithm. Each docking pose was evaluated by the following Dock Score energy function:

 Dock Score = − (ligand/receptor interaction energy + ligand internal energy).

### 2.3. Molecular Dynamics (MD) Simulation

The molecular dynamics (MD) simulation utilizing Gromacs 4.5.5 [50] was employed using classical molecular dynamics theory to simulate each protein-ligand complex under dynamic conditions. In preparation section, the IDH2 R140Q mutant proteins were prepared by pdb2gmx protocol of Gromacs to provide topology and parameters with charmm27 force field, and each ligand was prepared by SwissParam program [51]. A cubic box solvated using TIP3P water model was defined based upon the edge approx. 1.2 nm from the protein complexes periphery. In the minimization section, the steepest descent [52] minimization was employed with a maximum of 5,000 steps to remove bad van der Waals contacts. Gromacs program creates a neutral system using 0.145 M NaCl model, followed by another steepest descent minimization with a maximum of 5,000 steps to remove bad van der Waals contacts. In the equilibration section, Gromacs program performs a position-restrained molecular dynamics with the linear constraint algorithm for all bonds, NVT equilibration, Berendsen weak thermal coupling method, and particle mesh Ewald method. In the production section, Gromacs program performs a total of 5000 ps production simulation with time step in unit of 2 fs under NPT ensembles and particle mesh Ewald (PME) option. A series of protocols in Gromacs program was utilized to analyze the 5000 ps MD trajectories. The CAVER 3.0 [53] was employed to analyze the presumably pathways for small molecule under dynamics conditions.

## 3. Results and Discussion

### 3.1. Disordered Protein Prediction

**Figure 1:**
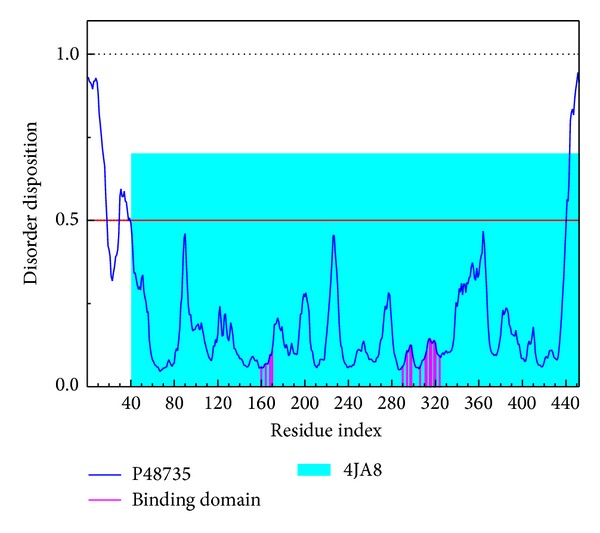
Disordered disposition predicted by PONDR-Fit.

The sequence of IDH2 protein from Swiss-Prot (UniProtKB: P48735) was employed to predict the disordered disposition by PONDR-Fit protocol. As illustrated in Figure 1, the key residues in the binding domain have no disordered disposition, which express a stable binding domain in protein folding. It indicates that the binding domain in the crystallography structure of target protein will be suitable for docking simulation as the residues in the binding domain have no significant variation.

### 3.2. Docking Simulation

**Figure 2:**
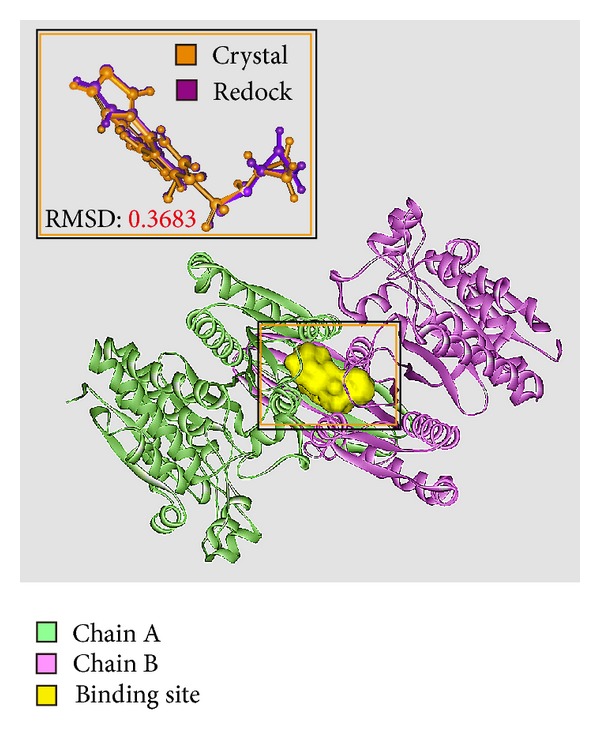
Binding site of IDH2 R140Q mutant protein defined as the volume of AGI-6780 and root-mean-square deviation value between crystallized structure (orange) and docking pose (violet) of AGI-6780.

**Figure 3:**
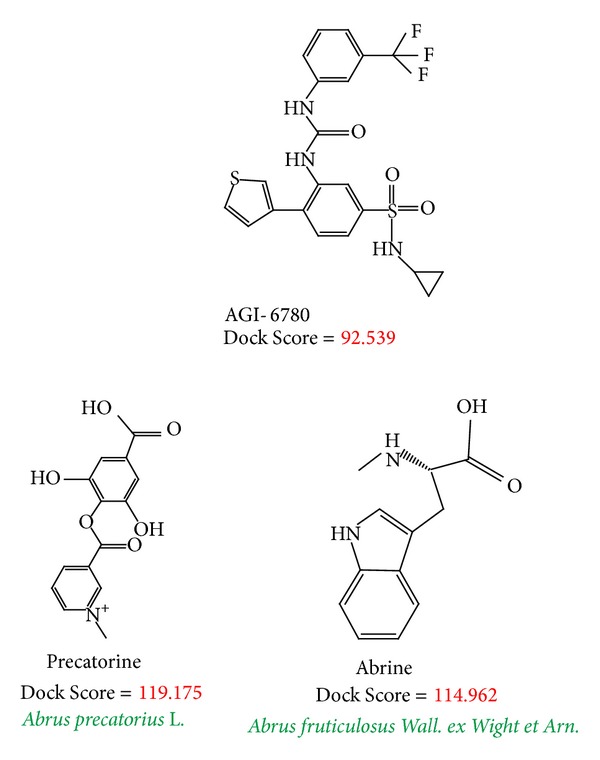
Chemical scaffold of controls and top two TCM candidates with their scoring function and sources.

**Figure 4:**
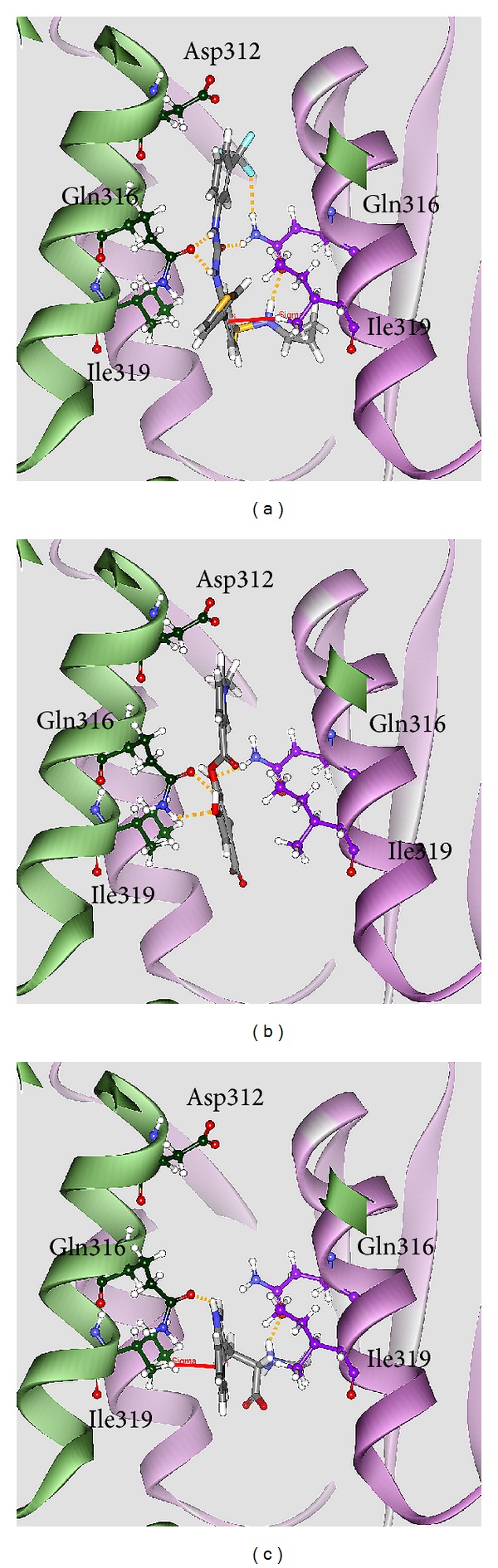
Docking pose of IDH2 R140Q mutant protein complexes with (a) AGI-6780, (b) precatorine, and (c) abrine.

To validate the accuracy of LigandFit protocol, we redock the cocrystallized IDH2 R140Q mutant inhibitor, AGI-6780, into the binding site of IDH2 R140Q mutant proteins. Root-mean-square deviation (RMSD) value between crystallized structure and docking pose of AGI-6780 is 0.3683 Å (Figure 2), which indicates that the docking simulation by LigandFit protocol is suitable for virtual screening with IDH2 R140Q mutant proteins. After virtual screening, the chemical scaffolds of AGI-6780 and top two TCM compounds are displayed in Figure 3 with Dock Score and sources. Precatorine is extracted from* Abrus precatorius* L., and abrine is extracted from* Abrus fruticuIosus Wall. ex Wight et Arn.* The compounds extracted from* Abrus precatorius* L. had been indicated to have the antimicrobial activity [54], antibacterial activity [55], cytotoxic and proapoptotic effects for breast cancer [56], and several tumor lines [57].  Figure 4 illustrated the docking poses of IDH2 R140Q mutant protein complexes with AGI-6780 and top two TCM compounds, respectively. The IDH2 R140Q mutant protein inhibitor, AGI-6780, has hydrogen bonds (H-bonds) with residues Gln316 in both chains of IDH2 R140Q mutant protein and a *π* interaction with residue Ile319 in chain B of IDH2 R140Q mutant protein. For the top TCM candidates, they also have H-bonds with residues Gln316 in both chains of IDH2 R140Q mutant protein as AGI-6780. For abrine, it has a *π* interaction with residue Ile319 in chain A of IDH2 R140Q mutant protein.

### 3.3. Molecular Dynamics Simulation

**Figure 5:**
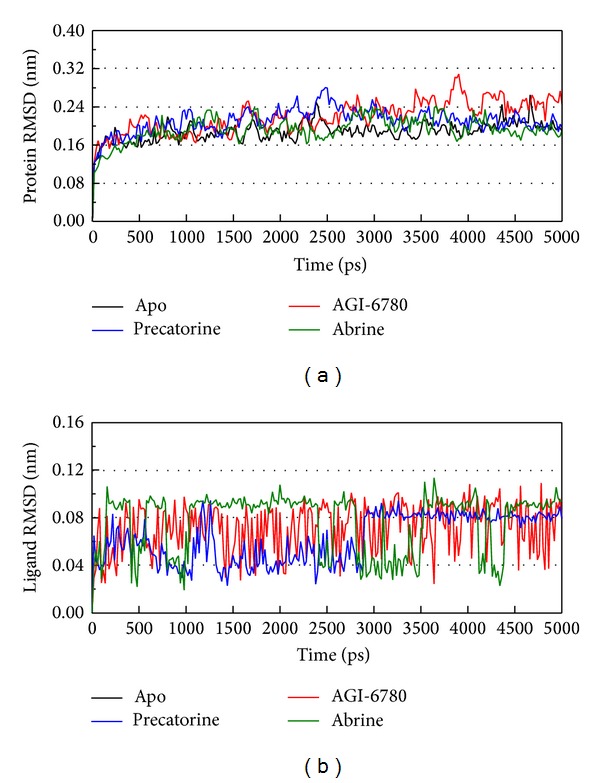
Root-mean-square deviations in units of nm for protein and ligand over 5000 ps of MD simulation for IDH2 R140Q mutant proteins and protein complexes with AGI-6780, precatorine, and abrine.

**Figure 6:**
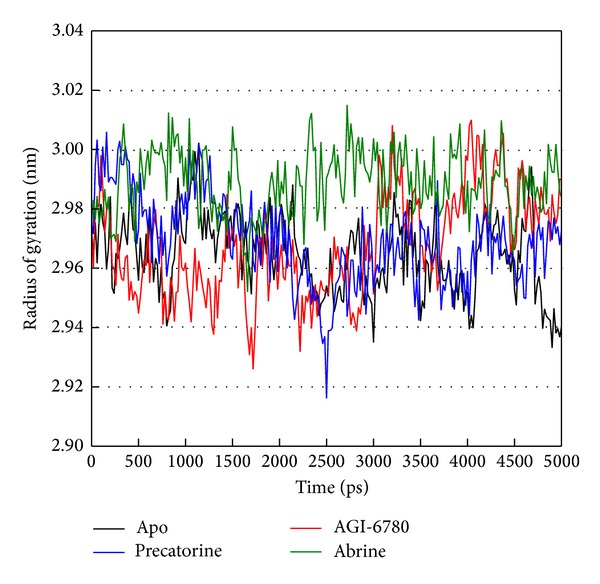
Radii of gyration for protein over 5000 ps of MD simulation for IDH2 R140Q mutant proteins and protein complexes with AGI-6780, precatorine, and abrine.

**Figure 7:**
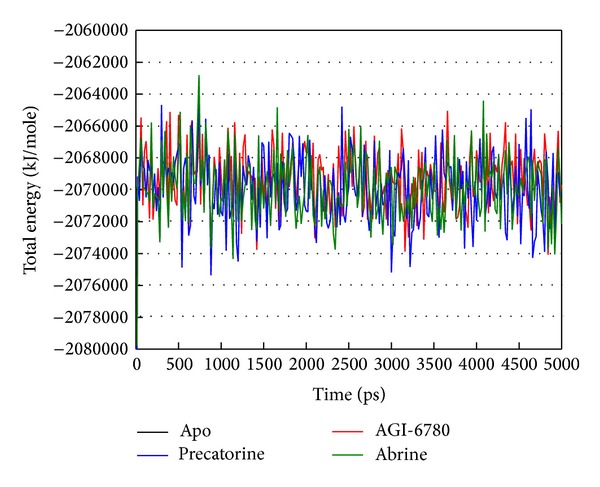
Variation of total energy for complex over 5000 ps of MD simulation for IDH2 R140Q mutant proteins and protein complexes with AGI-6780, precatorine, and abrine.

**Figure 8:**
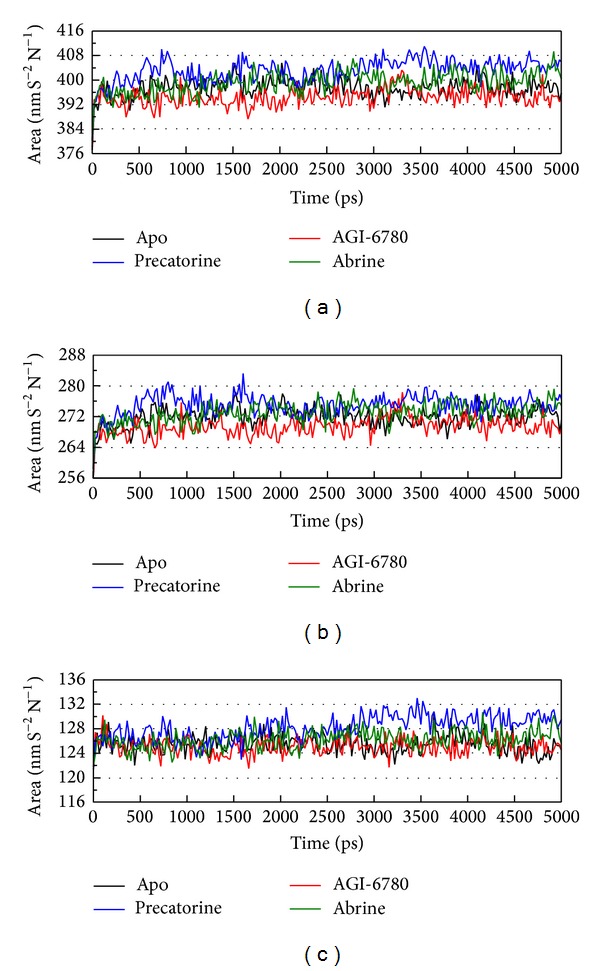
Variation of (a) total solvent accessible surface area, (b) hydrophobic surface area, and (c) hydrophilic surface area over 5000 ps of MD simulation for IDH2 R140Q mutant proteins and protein complexes with AGI-6780, precatorine, and abrine.

**Figure 9:**
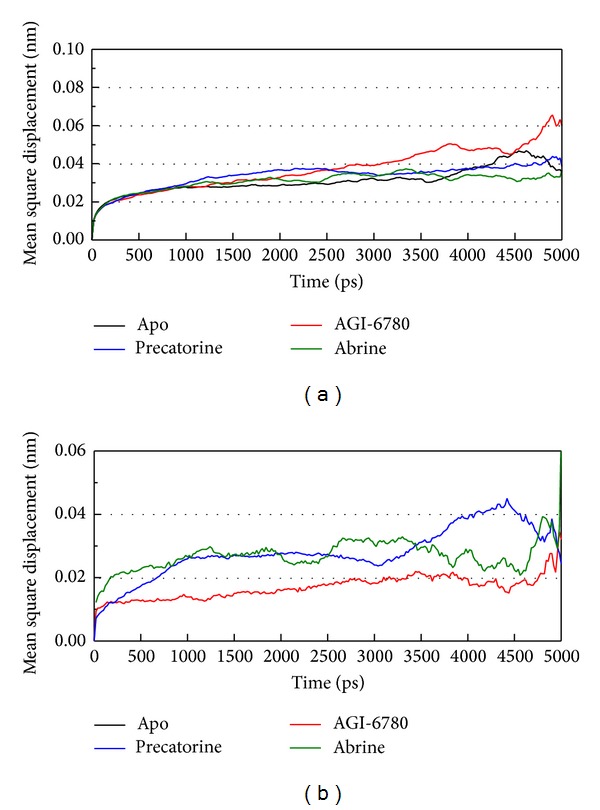
Mean square displacement (MSD) for (a) protein and (b) ligand over 5000 ps of MD simulation for IDH2 R140Q mutant proteins and protein complexes with AGI-6780, precatorine, and abrine.

**Figure 10:**
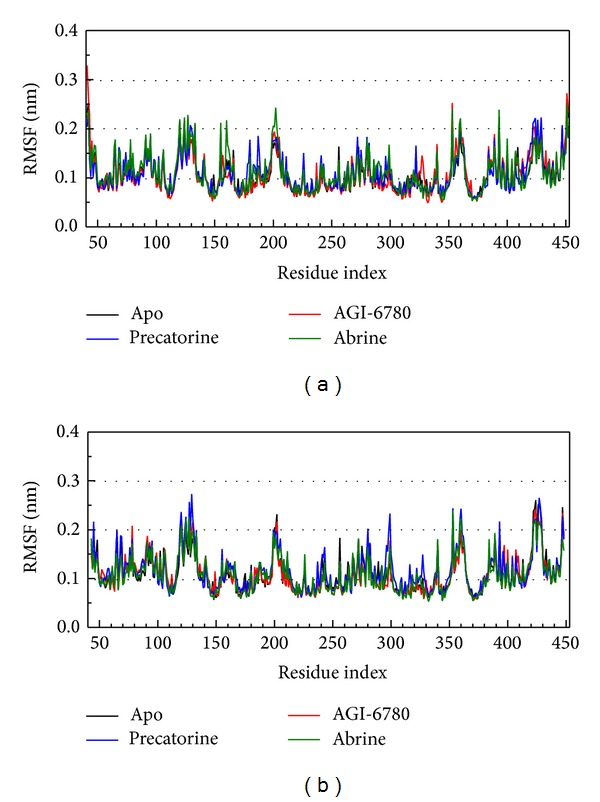
Root-mean-square fluctuation (RMSF) for residues in (a) chain A and (b) chain B of IDH2 R140Q mutant proteins and protein complexes with AGI-6780, precatorine, and abrine.

For the docking simulation performed by LigandFit protocol, the receptor is a rigid body of IDH2 R140Q mutant proteins. The conformation of the IDH2 R140Q mutant protein may modify under dynamic conditions. We employed the MD simulation to validate the stability of interactions between IDH2 R140Q mutant proteins and each ligand. RMSDs illustrated the atomic fluctuations during MD simulation.  Figure 5 displays the atomic fluctuations of IDH2 R140Q mutant proteins in apo form and complexes with AGI-6780, precatorine, and abrine and the atomic fluctuations of each compound during 5000 ps MD simulation. It shows that IDH2 R140Q mutant proteins tend to be stable after first 100 ps MD simulation, but the ligands except precatorine are fluctuate during MD simulation. To consider the variation radii of gyration for protein and total energy over 5000 ps MD simulation in Figure 6, it indicates that the radii of gyration for IDH2 R140Q mutant proteins in apo form were decreased after 4500 ps MD simulation, but the radii of gyration for complexes of IDH2 R140Q mutant proteins with AGI-6780, precatorine, and abrine were more stabilized. In addition, there is no significant change for the total energies of each IDH2 R140Q mutant protein complex during MD simulation in Figure 7. The variation of solvent accessible surface area over 5000 ps MD simulation in Figure 8 indicates that docking the ligands, AGI-6780, precatorine, and abrine, would not affect the solvent accessible surface of IDH2 R140Q mutant protein under dynamic conditions. The mean square displacement (MSD) for each protein and ligand in IDH2 R140Q mutant proteins and protein complexes with AGI-6780, precatorine, and abrine over 5000 ps of MD simulation is displayed in Figure 9. Root-mean-square fluctuation (RMSF) for each residue over 5000 ps MD simulation is displayed in Figure 10. They indicate that IDH2 R140Q mutant protein docking with precatorine and abrine causes similar diffusion constant and flexibility for IDH2 R140Q mutant proteins as AGI-6780.

**Figure 11:**
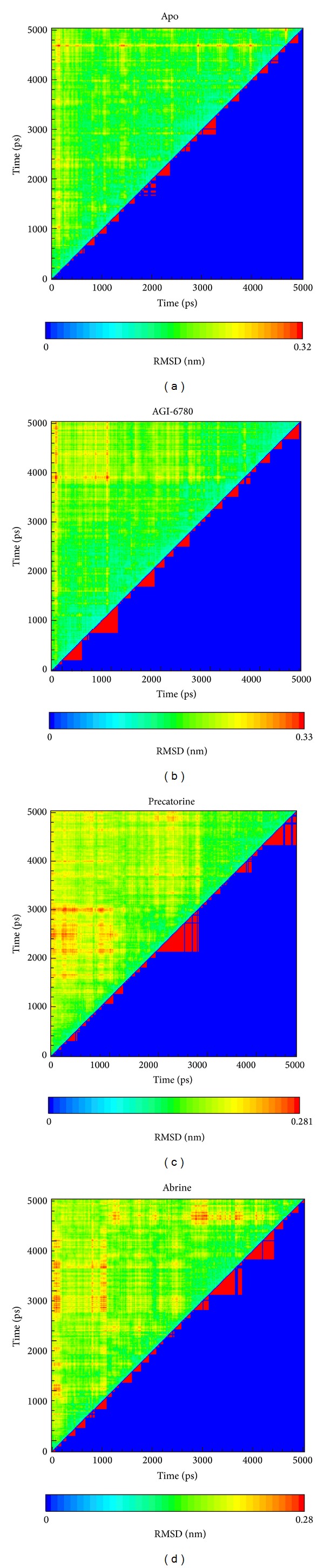
Root-mean-square deviation value (upper left half) and graphical depiction of the clusters with cutoff 0.105 nm (lower right half) for IDH2 R140Q mutant proteins and protein complexes with AGI-6780, precatorine, and abrine.

**Figure 12:**
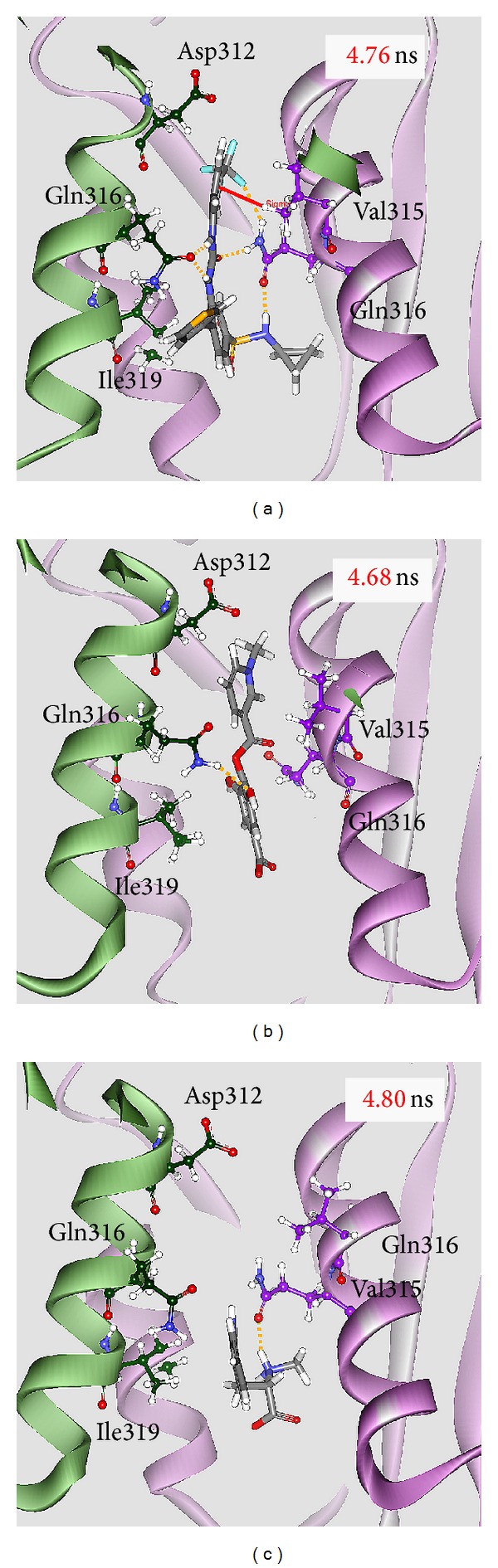
Docking poses of middle RMSD structure in the major cluster for IDH2 R140Q mutant protein complexes with AGI-6780, precatorine, and abrine.

**Figure 13:**
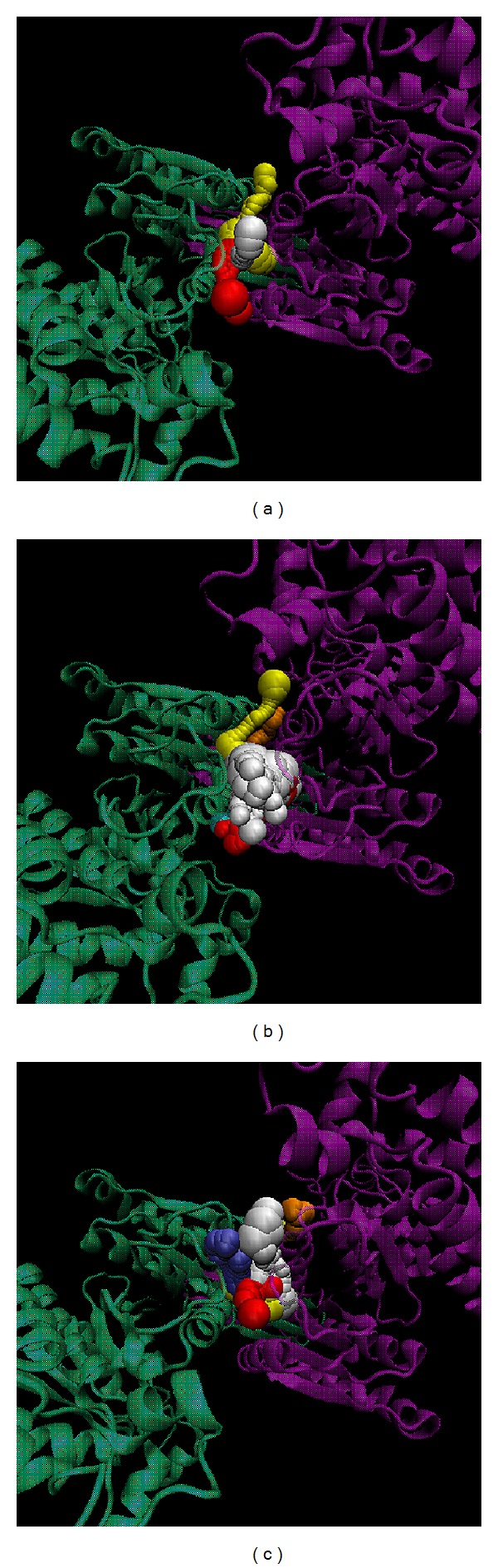
Analysis of transport pathways for IDH2 R140Q mutant protein complexes with (a) AGI-6780, (b) precatorine, and (c) abrine.

After MD simulation, we identify the representative structures of IDH2 R140Q mutant proteins in apo form and in each complex using the RMSD values and graphical depiction of the clusters analysis with a RMSD cutoff of 0.105 nm in Figure 11. The docking poses of the representative structures for complexes of IDH2 R140Q mutant proteins with AGI-6780, precatorine, and abrine are illustrated in Figure 12. To compare with the result in docking simulation, the IDH2 R140Q mutant protein inhibitor, AGI-6780, has stable hydrogen bonds (H-bonds) with residues Gln316 in both chains of IDH2 R140Q mutant protein and forms a *π* interaction with residue Val315 in chain B of IDH2 R140Q mutant protein. For TCM candidates, they have similar docking poses as docking simulation, which has stable H-bonds with residues Gln316. The H-bond occupancy for key residues in complexes of IDH2 R140Q mutant protein with AGI-6780 and top TCM compounds overall 5000 ps of molecular dynamics simulation in Table 1 displayed the stability of H-bonds. Analysis of transport pathways for each IDH2 R140Q mutant protein complex illustrated in Figure 13 shows the presumably pathways for small molecule. They indicate that IDH2 R140Q mutant protein docking with precatorine and abrine has similar effects of protein conformation as AGI-6780.

## 4. Conclusion

This study aims to investigate the potent lead TCM candidates for IDH2 R140Q mutant protein inhibitors against cancers. Compared to the IDH2 R140Q mutant protein inhibitor, AGI-6780, the top two TCM compounds, precatorine and abrine, have higher binding affinities with target protein in docking simulation. Both of them has H-bonds with residues Gln316 in both chains of IDH2 R140Q mutant protein as AGI-6780. After MD simulation, the top two TCM compounds remain as the same docking poses under dynamic conditions. In addition, precatorine is extracted from* Abrus precatorius* L., which has been indicated to have the cytotoxic and proapoptotic effects for breast cancer and several tumor lines. Hence, we propose the TCM compounds, precatorine and abrine, as potential candidates as lead compounds for further study in drug development process with the IDH2 R140Q mutant protein against cancer.

## Figures and Tables

**Table 1:** H-bond occupancy for key residues of IDH2 R140Q mutant protein with AGI-6780 and top TCM compounds overall 5000 ps of molecular dynamics simulation.

Name	H-bond interaction	Occupancy
AGI-6780	A:Gln316:OE1/H37	100%
	A:Gln316:OE1/H38	100%
	B:Gln316:HE22/O13	100%
	B:Gln316:OE1/H40	100%

Precatorine	A:Gln316:HE22/O20	67%
	A:Gln316:OE1/H29	14%
	B:Gln316:HE22/O9	4%
	B:Gln316:HE22/O16	12%
	B:Gln316:OE1/H28	4%

Abrine	B:Gln316:HE22/N7	22%
	B:Gln316:OE1/H30	95%
	B:Gln316:O/H30	2%

H-bond occupancy cutoff: 0.3 nm.
